# Do glycaemic treatment targets affect the perinatal mental health status of women with gestational diabetes? – Data from the TARGET Trial

**DOI:** 10.1186/s12884-023-06190-4

**Published:** 2023-12-16

**Authors:** Phyllis Ohene-Agyei, Thach Tran, Jane E. Harding, Caroline A. Crowther

**Affiliations:** 1 Liggins Institute, University of Auckland, Auckland, New Zealand; 2 School of Biomedical Engineering, University of Technology Sydney, Sydney, Australia

**Keywords:** Gestational diabetes mellitus, Cluster-randomized trial, Stepped-wedge design, Glycaemic treatment targets, Anxiety, Depression, Health-related quality of life

## Abstract

**Background:**

Gestational diabetes mellitus is associated with perinatal mental disorders. Effective management may reduce this risk, but there is little evidence on effects of different glycaemic treatment targets. We assessed whether tight glycaemic treatment targets compared with less-tight targets reduce the risk of poor mental health outcomes in women with gestational diabetes.

**Methods:**

This was a secondary analysis of data from women who consented to complete perinatal mental health questionnaires as participants in the TARGET Trial, a stepped-wedge cluster randomized trial in 10 hospitals in New Zealand. All hospitals initially used less tight glycaemic targets for management of gestational diabetes and were sequentially randomized, in clusters of two at 4-monthly intervals, to using tighter glycaemic targets.

Data were collected from 414 participants on anxiety (6-item Spielberger State Anxiety scale), depression (Edinburgh Postnatal Depression Scale), and health-related quality of life (36-Item Short-Form General Health Survey) at the time of diagnosis (baseline), 36 weeks of gestation, and 6 months postpartum. The primary outcome was composite poor mental health (any of anxiety, vulnerability to depression, or poor mental health-related quality of life). Generalized linear mixed models were used to determine the main treatment effect with 95% confidence intervals using an intention-to-treat approach.

**Results:**

We found no differences between randomised glycaemic target groups in the primary outcome at 36 weeks’ (relative risk (RR): 1.07; 95% confidence interval 0.58, 1.95) and 6 months postpartum (RR: 1.03; 0.58, 1.81). There were similarly no differences in the components of the primary outcome at 36 weeks’ [anxiety (RR: 0.85; 0.44, 1.62), vulnerability to depression (RR: 1.10; 0.43, 2.83), or poor mental health-related quality of life (RR: 1.05; 0.50, 2.20)] or at 6 months postpartum [anxiety (RR:1.21; 0.59, 2.48), vulnerability to depression (RR:1.41; 0.53, 3.79), poor mental health-related quality of life (RR: 1.11; 0.59, 2.08)].

**Conclusion:**

We found no evidence that adoption of tighter glycaemic treatment targets in women with gestational diabetes alters their mental health status at 36 weeks’ gestation and at 6 months postpartum.

**Trial registration:**

The Australian New Zealand Clinical Trials Registry (ANZCTR). ACTRN12615000282583 (ANZCTR—Registration). Date of registration: 25 March 2015.

## Background

Gestational diabetes mellitus (GDM) refers to hyperglycaemia detected during pregnancy where glucose concentrations are above normal but below the diagnostic criteria of diabetes mellitus [1] GDM is the commonest metabolic diseases experienced in pregnancy, with an estimated one in six pregnant women affected globally [2]. GDM prevalence is increasing worldwide, with New Zealand experiencing an annual 14% increase in national prevalence from 2001 to 2012 [3], and the United Sates reporting an increase from 0.3% in 1979–81 to 5.8% in 2008–10 [4]. GDM can cause serious short and long term maternal and offspring complications, including poor maternal psychological outcomes. Results from two longitudinal studies indicated that women who had a diagnosis of GDM were 2–4 times more likely to experience perinatal depression compared with women without GDM [5, 6]. Two systematic reviews including observational and intervention studies reported that GDM diagnosis doubled the risk of antenatal depression and increased the risk of postnatal depression by more than 60% [7, 8]. Other psychological outcomes including anxiety and stress have also been reported to be higher among women with GDM compared to women without GDM [9–11]. Women with a concurrent diagnosis of GDM and antenatal depression have higher rates of poor perinatal outcomes including gestational hypertension, pre-eclampsia, preterm birth, and neonatal respiratory distress compared with those with GDM without depression [12, 13]. Potential mechanisms mediating this increased risk include biological, psychological, and environmental changes associated with depression, the pregnancy state and GDM management [14–16].

Poor glycaemic control has been associated with depression in people with diabetes outside of pregnancy [17]. However, in women with GDM, evidence on this is limited. The Australian Carbohydrate Intolerance Study in Pregnant Women (ACHOIS) Trial reported lower rates of depression and improved maternal health-related quality of life three months post-partum for women with GDM who received treatment compared with those with GDM who only received routine antenatal care [18]. That trial used glycaemic targets for management of GDM that were previously recommended in New Zealand (fasting plasma glucose < 5.5 mmol/L (< 99 mg/dl); 1-h postprandial < 8.0 mmol/L (< 144 mg/dl); 2-h postprandial < 7.0 mmol/L (< 126 mg/dl)) [19]. There has since been an international trend towards recommending tighter glycaemic targets (fasting plasma glucose ≤ 5.0 mmol/L (≤ 90 mg/dl), 1-h postprandial ≤ 7.4 mmol/L (≤ 133 mg/dl); 2-h postprandial ≤ 6.7 mmol/L (≤ 121 mg/dl)) for management of GDM [20, 21]. A 2016 systematic review of optimal glycaemic treatment targets for GDM concluded from observational studies that there were greater maternal and neonatal metabolic benefits when a fasting plasma glucose target of < 5.0 mmol/L was used [22]. The effect of these tighter glycaemic targets on maternal psychological well-being is, however, not known. The review highlighted the need for high-quality clinical trial evidence on different glycaemic targets and their impact on maternal and neonatal well-being.

To provide high quality evidence and based on recommendations from the New Zealand Ministry of Health [3], the TARGET Trial assessed the effect of different glycaemic treatment targets on maternal and infant health [23]. This study reports on the maternal mental health outcomes. We aimed to assess if tighter glycaemic treatment targets compared with less-tight targets reduced the risk of adverse mental health outcomes, namely anxiety, depression, and poor health-related quality of life in women with GDM.

## Methods

This study was nested within the TARGET Trial, a nationally representative multi-center stepped-wedge cluster randomised trial in 10 publicly funded participating hospitals in New Zealand [24]. Participating hospitals were cluster randomised and the intervention of tighter targets (fasting plasma glucose ≤ 5.0 mmol/L ( ≤ 90 mg/dl), 1-h postprandial ≤ 7.4 mmol/L ( ≤ 133 mg/dl); 2-h postprandial ≤ 6.7 mmol/L ( ≤ 121 mg/dl) sequentially implemented at 4-monthly intervals in place of the less-tight targets (fasting plasma glucose < 5.5 mmol/L (< 99 mg/dl); 1-h postprandial < 8.0 mmol/L (< 144 mg/dl); 2-h postprandial < 7.0 mmol/L (< 126 mg/dl). The allocation sequence of the hospitals to the implementation of the tight glycaemic targets was prepared by a statistician using a computer-generated random number table. Women diagnosed with GDM were treated based on the targets being used by the hospital at the time they received their antenatal care. The care the women in our study received were guided by the New Zealand guidelines for management of gestational diabetes [3]. The guideline recommends specialised dietary and lifestyle advice, and medication if required to achieve glycaemic treatment targets. Postpartum follow up care is recommended at 3 months after birth for glucose screening. Health professionals involved in their care included their lead maternity carers (midwives and obstetricians) and health professionals of their local Diabetes Pregnancy Service (diabetes specialists, diabetes nurses, and dieticians). Women were blinded to their glycaemic target groups as per the study protocol [24].

Women who were recruited to the TARGET Trial were invited to participate in this nested study and complete questionnaires about self-reported depression, anxiety, and health-related quality of life (HRQoL) at the time of diagnosis of GDM (baseline), 36 weeks’ gestation, and 6 months after the birth. Questionnaires were provided by designated study health professionals in each hospital and the women independently completed them and returned them during their clinic appointments or by post. The TARGET Trial was registered with the Australian New Zealand Clinical Trials Registry—ACTRN 12615000282583. Human ethics approval was granted by the Northern A Health and Disability Ethics Committee in New Zealand (14/NTA/163/AMO1). All participants provided written informed consent for participation in this nested study.

### Outcomes

The primary outcome of this study was the proportion of women with a composite of poor mental health outcomes (defined as any of vulnerability to depression, anxiety, or poor mental HRQoL) at 36 weeks’ gestation and 6 months after birth.

Depression was measured using the Edinburgh Postnatal Depression Scale (EPDS), a validated tool for assessing postpartum depression among pregnant women [25]. The tool comprises 10 items with each item scored on a 4-point scale (0 – 3) for a maximum score of 30. A cut-off score of > 12 indicates significant vulnerability to depression [18, 26]. Anxiety was measured using the shortened 6-item Spielberger State-Trait Anxiety Inventory (STAI) [27]. The STAI accurately reflects the anxiety‐related experiences of pregnant women [28]. In our study we used the shortened form (6-item STAI) which has been shown as a valid alternative to the full version for use in research as it improves acceptability while maintaining its validity [27].The tool includes 6 items using a 4-point Likert-type scale (1 = *not at all* to 4 = *very much so*), with scores > 15 indicating presence of symptoms of anxiety [29, 30] as used in similar studies [18]. HRQoL was assessed using the 36-Item Short-Form General Health Survey (SF-36), a validated tool for assessing quality of life measures during pregnancy [31]. The tool uses 36 items to assess eight aspects of health status: general health, mental health, physical functioning, social functioning, role physical, role emotional, bodily pain, and vitality [32]. The scores range from 0–100 and two summary measures, namely physical component summary (PCS) and mental component summary (MCS), can also be calculated [33] with higher scores being associated with higher levels of functioning. We assigned a cut-off value of MCS < 40 (less than minus one standard deviation from the New Zealand standardized mean of 50) [34] to denote poor mental HRQoL, as this measure adequately captures mental health outcomes [35]. This cut-off has good positive predictive value for poor mental health outcomes compared to other validated psychological instruments [36].

Secondary outcomes assessed included incidence of anxiety, depression, and poor mental HRQoL at 36 weeks and 6 months postpartum, and mean STAI, EPDS, and SF-36 (all eight scales of the SF-36 and the two summary measures) scores at 36 weeks’ gestation and 6 months postpartum.

### Statistical analysis

Baseline characteristics of the participants were compared between the two glycaemic target groups using student’s t-tests or chi-squared tests where appropriate. Psychological outcomes were analyzed both as continuous and categorical variables to enhance clinical interpretations using the intention-to-treat approach [37, 38]. Generalised linear mixed models were used to determine the main treatment effect with random effect for hospitals, and fixed effects for the intervention, and time interval between initiation of the assigned target and GDM diagnosis. The analyses were adjusted for predefined confounding effect of gestational age at trial entry, body mass index (BMI), ethnicity, and history of GDM. Binary outcomes were analysed using a log Poisson mixed-effects regression with robust variance estimation and the treatment effect was reported as relative risk and 95% confidence interval (CI). A linear mixed-effects regression was conducted to analyse continuous outcomes with further adjustment for their value at the study entry to obtain the mean difference and 95% CI. No adjustments were made for multiple comparisons. A 2-sided p-value < 0.05 was considered statistically significant. Statistical analyses were conducted using SAS software version 9.4 (SAS Institute, Cary, North Carolina, United States of America).

## Results

### Baseline characteristics and outcome measures of the study population

**Fig. 1:**
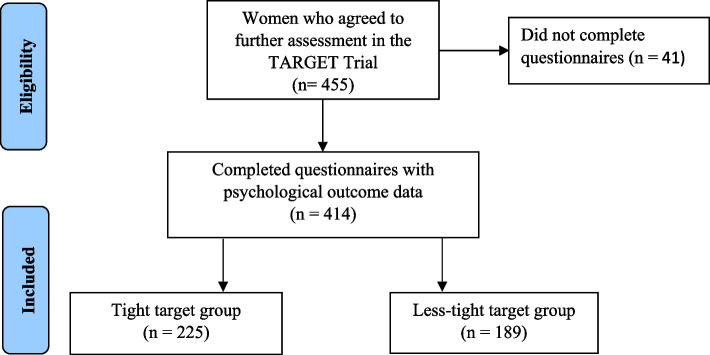
Study eligibility and inclusion. Figure shows the eligibility and inclusion of women in our study from the TARGET Trial

Women were recruited to the TARGET trial between May 29, 2015 and November 7, 2017. Of the 455 eligible women, 414 completed the psychological questionnaires (Fig. 1). Of those, 225 (54.3%) women were randomised to the tighter glycemic targets and 189 (45.7%) to the less tight targets. Baseline characteristics were generally similar among women in the two treatment groups (Table 1). However, there were more European women in the less-tight treatment group and more Pacific women in the tighter treatment group. Most of the women in the study were overweight or obese (90%), with more obese women in the tighter target group.

**Table 1:** Baseline characteristics of women randomized to tight or less tight glycaemic targets

Characteristics	Tight glycaemic targets (*n* = 225)	Less-tight glycaemic targets (*n* = 189)	Total (*N* = 414)
Age (years)	32.5 ± 5.0	32.8 ± 5.2	32.7 ± 5.1
Maternal ethnicity			
NZ European	76 (33.8)	104 (55.0)	180 (43.5)
Māori	27 (12.0)	16 (8.5)	43 (10.4)
Pacific	42 (18.7)	15 (7.9)	57 (13.8)
Asian	77 (34.2)	50 (26.5)	127 (30.7)
Other	3 (1.3)	4 (2.1)	7 (1.7)
BMI (kg/m^2^):			
18.5–24.9 (normal)	20 (9.0)	21 (11.2)	41 (10.0)
25.0–29.9 (overweight)	64 (28.7)	63 (33.5)	127 (30.9)
≥ 30 (obese)	139 (62.3)	104 (55.3)	243 (59.1)
Smoked in pregnancy	16 (7.1)	15 (7.9)	31 (7.5)
Weight at entry (kg)	86.9 ± 21.6	86.4 ± 21.9	86.7 ± 21.7
Height at entry (cm)	162.7 ± 6.7	162.3 ± 7.0	162.5 ± 6.8
Primiparous	93 (41.3)	81 (42.9)	174 (42.0)
Any previous perinatal death^a^	12 (9.1)	1 (0.9)	13 (5.4)
Previous GDM^a^			
Yes	37 (28.0)	41 (38.0)	78 (32.5)
No	94 (71.2)	66 (61.1)	160 (66.7)
Unknown	1(0.8)	1 (0.9)	2 (0.8)
OGTT (mmol/L)			
Fasting	5.0 (4.6–5.6)	5.0 (4.4–5.7)	5.0 (4.5–5.7)
1-h postprandial	10.3 (10.0–11.8)	11.2 (10.2–12.7)	11.1 (10.2–12.2)
2-h postprandial	9.4 (9.0–10.1)	9.5 (9.0–10.3)	9.4 (9.0–10.1)
Blood pressure (mmHg)			
Systolic	110.0 (100.0–118.0)	110.0 (102.0–120.0)	110.0 (100.0–120.0)
Diastolic	68.0 (60.0–72.0)	68.0 (60.0–75.0)	68.0 (60.0–74.0)

^a^Among women with previous pregnancy of 20 weeks gestation or more

All variables presented as mean ± standard deviation, number (percentage), or median (interquartile range) unless otherwise indicated

*BMI* body mass index, *GA* gestational age, *GDM* gestational diabetes mellitus, *OGTT* oral glucose tolerance test, *SD* standard deviation

At entry into the trial there were no differences in mental health composite outcome or its components between the two target groups (Table 2). The tighter target group had higher scores in the vitality domain of the SF-36 (54.5 ± 14.4 vs 51.5 ± 14.7, p = 0.04), but there were no significant differences between groups in the other domains or in the overall summary scores.

**Table 2:** Mental health measures at trial entry (baseline)

Outcome	Tight glycaemic targets (*n* = 225)	Less-tight glycaemic targets (*n* = 189)	*p*-value
Poor mental health composite^a^	60/214 (28.0)	45/182 (24.7)	0.46
Anxiety (STAI > 15)	44/219 (20.1)	27/189 (14.3)	0.12
STAI score	11.0 ± 3.4	11.0 ± 3.6	0.92
Depression (EPDS > 12)	34/220 (15.5)	32/185 (17.3)	0.62
EPDS score	7.6 ± 4.9	7.6 ± 4.6	0.98
Poor mental HRQoL (MCS < 40)	36/220 (16.4)	32/184 (17.4)	0.80
SF-36 scores			
General health	69.9 ± 18.8	70.6 ± 16.7	0.68
Mental health	68.1 ± 11.8	68.1 ± 12.4	0.94
Physical functioning	60.9 ± 22.8	64.5 ± 20.9	0.09
Social functioning	73.8 ± 23.4	76.3 ± 23.7	0.29
Role physical	49.1 ± 39.8	53.8 ± 40.1	0.23
Role emotional	77.7 ± 36.0	82.5 ± 32.7	0.16
Bodily pain	63.2 ± 22.6	64.5 ± 21.4	0.53
Vitality	54.5 ± 14.4	51.5 ± 14.7	0.04
PCS	41.5 ± 9.5	42.8 ± 8.2	0.14
MCS	48.5 ± 7.7	48.5 ± 8.4	0.99

^a^Any of anxiety (STAI > 15), vulnerability to depression (EPDS > 12) or poor mental health-related quality of life (SF-36 MCS < 40)

All variables presented as number (percentage) or mean ± standard deviation unless otherwise indicated

*EPDS* Edinburgh Postnatal Depression Scale, *HRQoL* health-related quality of life, *MCS* Mental Component Summary, *PCS* Physical Component Summary, *STAI* Spielberger State-Trait Anxiety Inventory, *SD* standard deviation, *SF-36* 36-Item Short-Form General Health Survey

### Incidence of anxiety, depression and poor mental HRQoL at 36 weeks’ gestation and 6 months after birth

After adjustment for potential confounders, there were no differences between the two treatment groups at 36 weeks’ gestation in the composite of poor mental health outcomes [adjusted relative risk (aRR) 1.07 (95% CI 0.58, 1.95)] or its components [anxiety: aRR 0.85 (0.44, 1.62), vulnerability to depression: aRR: 1.10 (0.43, 2.83), poor mental HRQoL: aRR: 1.05 (0.50, 2.20)]. Similarly, there was no difference in mean scores for anxiety [adjusted mean difference (aMD) (95% CI) 0.00 (-0.80, 0.81)], depression [-0.12 (-0.90, 0.66)] and HRQoL MCS [0.20 (-1.36, 1.76)] (Table 3).

**Table 3:** Comparison of mental health outcomes at 36 weeks’ gestation

Outcome	Tight glycaemic targets		Less-tight glycaemic targets		Relative risk or mean difference (95% CI)			
	N	n (%) or mean ± SD	N	n (%) or mean ± SD	Unadjusted	*p*-value	Adjusted^b^	*p*-value
Poor mental health composite^a^	168	39 (23.2)	153	26 (17.0)	1.25 (0.69, 2.27)	0.45	1.07 (0.58, 1.95)	0.84
Anxiety (STAI > 15)	171	23 (13.5)	156	27 (17.3)	0.87 (0.45, 1.69)	0.67	0.85 (0.44, 1.62)	0.62
STAI score	171	10.6 ± 3.4	156	10.9 ± 3.6	0.01 (-0.89, 0.91)	0.99	0.00 (-0.80, 0.81)	0.99
Depression (EPDS > 12)	180	16 (8.9)	154	11 (7.1)	1.36 (0.55, 3.35)	0.51	1.10 (0.43, 3.83)	0.85
EPDS score	180	6.4 ± 4.2	154	6.4 ± 4.5	0.01 (-1.11, 1.12)	0.99	-0.12 (0.90, 0.66)	0.77
Poor mental HRQoL (MCS < 40)	175	26 (14.9)	157	17 (10.8)	1.13 (0.54, 2.37)	0.74	1.05 (0.50, 2.20)	0.90
SF-36 scores:								
General health	178	71.2 ± 17.8	157	71.9 ± 17.1	0.48 (-4.01, 4.96)	0.83	-0.37 (-3.79, 3.04)	0.83
Mental health	182	69.0 ± 12.0	157	70.6 ± 10.5	-1.16 (-4.06, 1.74)	0.43	-0.20 (-2.62, 2.23)	0.87
Physical functioning	182	58.1 ± 22.1	157	59.4 ± 22.3	-1.96 (-7.62, 3.70)	0.50	-0.86 (-5.80, 4.08)	0.73
Social functioning	183	75.0 ± 22.6	157	76.6 ± 21.4	-2.26 (-7.91, 3.39)	0.43	-1.03 (-5.79, 3.74)	0.67
Role physical	182	47.0 ± 42.1	157	44.6 ± 40.8	-7.0 (-11.30, 9.90)	0.90	2.53 (-6.92, 11.98)	0.60
Role emotional	182	77.2 ± 37.1	157	77.9 ± 35.4	0.08 (-9.23, 9.40)	0.99	1.44 (-6.88, 9.75)	0.73
Bodily pain	183	60.3 ± 22.4	157	60.1 ± 20.7	-0.27 (-5.83, 5.29)	0.92	1.26 (-3.41, 5.93)	0.60
Vitality	179	55.3 ± 14.3	157	51.2 ± 14.5	2.44 (-1.35, 6.23)	0.21	0.82 (-1.89, 3.53)	0.55
PCS	175	40.2 ± 8.9	157	40.0 ± 9.2	-2.0 (-2.53, 2.14)	0.87	0.42 (-1.49, 2.33)	0.67
MCS	175	49.4 ± 7.7	157	49.8 ± 7.1	0.04 (-1.88, 1.96)	0.97	0.20 (-1.36, 1.76)	0.80

Any of anxiety (STAI > 15), vulnerability to depression (EPDS > 12) or poor mental health-related quality of life (SF-36 MCS < 40)

^b^ Adjusted for body mass index, gestational age at oral glucose tolerance test, ethnicity, and history of gestational diabetes

*CI* confidence interval, *EPDS* Edinburgh Postnatal Depression Scale, *HRQoL* health-related quality of life, *MCS* Mental Component Summary, *PCS* Physical Component Summary, *RR* Relative risk, *STAI* Spielberger State-Trait Anxiety Inventory, *SD* standard deviation, *SF-36* 36-Item Short-Form General Health Survey

At 6 months post-partum there were no differences between the treatment target groups for the composite of poor mental health outcomes [aRR 1.03 (0.58, 1.81)] or its components [anxiety: aRR 1.21 (0.59, 2.48), vulnerability to depression: aRR 1.41 (0.53, 3.79), poor mental HRQoL: aRR 1.11 (0.59, 2.08)]; or in scores for anxiety [aMD (95%CI) -0.05 (-0.86,0.76)], depression [0.47 (-0.52, 1.45)] and HRQoL MCS [0.44 (-1.60, 2.48)] (Table 4).

**Table 4:** Comparison of mental health outcomes at 6 months postpartum

Outcome	Tight glycemic targets		Less-tight glycemic targets		Relative risk or mean difference (95% CI)			
	N	n (%) or mean ± SD	N	n (%) or mean ± SD	Unadjusted	*p*-value	Adjusted^b^	*p*-value
Poor mental health composite^a^	156	34 (21.8)	169	33 (19.5)	1.11 (0.63, 1.94)	0.72	1.03 (0.58, 1.81)	0.93
Anxiety (STAI > 15)	161	22 (13.7)	172	20 (11.6)	1.34 (0.67, 2.69)	0.41	1.21 (0.59, 2.48)	0.61
STAI score	161	10.0 ± 3.4	172	10.3 ± 3.5	-0.04 (-0.90, 0.81)	0.92	-0.05 (-0.86, 0.76)	0.90
Depression (EPDS > 12)	162	15 (9.3)	170	10 (5.9)	1.54 (0.62, 3.87)	0.36	1.41 (0.53, 3.79)	0.50
EPDS score	162	5.4 ± 4.9	170	5.3 ± 4.3	0.43 (-0.73, 1.58)	0.47	0.47 (-0.52, 1.45)	0.35
Poor mental HRQoL (MCS < 40)	159	28 (17.9)	171	26 (15.2)	1.22 (0.66, 2.28)	0.52	1.11 (0.59, 2.08)	0.75
SF-36 scores:								
General health	164	72.6 ± 19.4	172	73.8 ± 17.7	-0.98 (-5.61, 3.66)	0.68	-0.48 (-4.71, 3.76)	0.83
Mental health	165	72.3 ± 12.3	172	70.6 ± 12.1	0.57 (-2.56, 3.70)	0.72	-0.25 (-3.08, 2.58)	0.86
Physical functioning	164	83.4 ± 23.4	172	89.1 ± 18.5	-3.84 (-9.18, 1.50)	0.16	-3.20 (-8.44, 2.05)	0.23
Social functioning	166	85.4 ± 21.4	172	85.5 ± 19.4	0.13 (-4.98, 5.24)	0.96	1.89 (-2.98, 6.76)	0.45
Role physical	164	83.7 ± 31.8	171	90.5 ± 22.6	-5.93 (-13.03, 1.17)	0.10	-2.29 (-9.18, 4.61)	0.52
Role emotional	164	84.6 ± 32.9	171	89.7 ± 25.9	-5.49 (-12.94, 1.96)	0.15	-2.42 (-9.77, 4.92)	0.52
Bodily pain	166	76.1 ± 21.5	172	78.6 ± 22.2	-2.34 (-7.80, 3.13)	0.40	-0.51 (-5.86, 4.84)	0.85
Vitality	165	58.9 ± 15.5	172	57.4 ± 14.7	1.12 (-2.95, 5.19)	0.59	0.06 (-3.17, 3.30)	0.97
PCS	159	51.0 ± 8.6	171	53.4 ± 7.4	-1.40 (-3.44, 0.65)	0.18	-0.83 (-2.84, 1.19)	0.42
MCS	159	48.3 ± 9.2	171	47.2 ± 7.6	0.60 (-1.52, 2.72)	0.58	0.44 (-1.60, 2.48)	0.67

^a^Any of anxiety (STAI > 15), vulnerability to depression (EPDS > 12) or poor mental health-related quality of life (SF-36 MCS < 40)

^b^Adjusted for body mass index, gestational age at oral glucose tolerance test, ethnicity, and history of gestational diabetes

*CI* confidence interval, *EPDS* Edinburgh Postnatal Depression Scale, *HRQoL* health-related quality of life, *MCS* Mental Component Summary, *PCS* Physical Component Summary, *RR* Relative risk, *STAI* Spielberger State-Trait Anxiety Inventory, *SD* standard deviation, *SF-36* 36-Item Short-Form General Health Survey

## Discussion

We found no significant differences in the proportion of women with anxiety, vulnerability for depression and poor mental HRQoL among women in the two glycaemic target groups. The mean scores for the measures of anxiety, depression and HRQoL were also not different between the two groups.

Currently recommended glycaemic treatment targets for the management of GDM differ across countries and professional associations. In the United States, the American Diabetes Association recommends fasting plasma glucose < 95 mg/dL (< 5.3 mmol/L); 1-h postprandial < 140 mg/dL (< 7.8 mmol/L); and 2-h postprandial < 120 mg/dL (< 6.7 mmol/L) [39], the National Institute for Health and Care Excellence (NICE) in the United Kingdom recommends a fasting plasma glucose < 5.3 mmol/L (< 95 mg/dL); 1 h postprandial < 7.8 mmol/L (< 140 mg/dL); and 2-h postprandial < 6.4 mmol/L (< 126 mg/dl) [40], and the World Health Organization a fasting plasma glucose ≤ 7.0 mmol/L (≤ 126 mg/dl); and 2-h postprandial ≤ 9.0 mmol/L (≤ 160 mg/dl) [41]. Most of these recommendations are not based on evidence from clinical trials but rather guideline panel consensus, with the relevant Cochrane systematic review reporting insufficient evidence on optimal glycemic targets to minimise adverse maternal and fetal health outcomes [42].

Studies assessing the association between glycaemic control and mental well-being in the general diabetes population have produced varying results. While some studies have showed good mental well-being is associated with intense glycaemic control [17, 43, 44], a few studies have reported the inverse (i.e., better mental well-being in women with poor glycaemic control) [45–47]. One of the reasons suggested for the latter finding is that more intense glycaemic control may require adherence to strict treatment practices, including dietary changes and addition of medication, which may cause anxiety and depression leading to lower mental well-being.

In women with GDM, evidence of a relationship between glycaemic control with psychological well-being is limited. In the TARGET Trial, women who were allocated to the tighter treatment target group had higher rates of use of pharmacological agents compared to those managed with the less-tight targets [23]. However, this does not seem to have resulted in poorer mental health outcomes. One cohort study of 68 women with GDM in the United States found no differences in psychological well-being between women who were diet-controlled compared with those who required insulin in addition to dietary therapy (intensive control) [48]. That study suggested that since blood glucose concentrations in women with GDM were less labile than in non-insulin dependent diabetics, the population amongst whom most studies have been conducted, this could explain the lack of association between mental health well-being and plasma glucose concentrations.

The improvement in maternal mental health outcomes found in the Australian Carbohydrate Intolerance Study in Pregnant Women (ACHOIS) trial has been suggested to represent the beneficial effect of increased care that women with GDM may receive when treatment is offered [49]. The lack of benefit or harm found in our study suggests additional care and monitoring generally associated with more intense management, as might be expected with tighter treatment targets, does not appear to either distress or reassure mothers.

Some studies have suggested that the link between GDM and poor mental health outcomes, especially depression, may be bidirectional i.e., depression may also precede GDM [14, 49, 50]. In our study, the incidence of vulnerability to depression in both treatment groups at baseline and 36 weeks’ gestation were lower than estimates from similar developed countries in the second and third trimesters [51], making this explanation unlikely in this cohort.

Additionally, some studies have suggested depression is associated with poor perceived glycaemic control in the general diabetes population [52, 53]. In our study, it may be that women in both treatment groups had a good perceived glycaemic control (albeit at different treatment targets) and hence showed no difference in the self-reported mental health outcomes. However, we had limited data on compliance to glycaemic treatment targets in our study and therefore could not explore this assumption further.

The incidence of depression and anxiety were similar between the two treatment groups at baseline, 36 weeks’ gestation, and 6 months postpartum. However, the incidence of depression and anxiety in both groups decreased at 36 weeks’ gestation compared to baseline. This result differs from some previous studies which reported a similar incidence of poor psychological outcome in the second and third trimesters in women with diabetes [54, 55]. However, it is consistent with other studies [56, 57], that reported better HRQoL scores among women with GDM at 36 weeks’ gestation compared to 6 months after birth. In comparison to the general population of pregnant women in New Zealand, the incidence of depression in our study in both treatment groups at 36 weeks’ gestation was lower than that estimated in pregnant women in their third trimester using nationally representative data (8.9% and 7.1% for tight target and less-tight target groups versus 11.9% using data from the Growing Up in New Zealand cohort) [58].

### Strengths and limitations

This study has several strengths. The data are from a randomised trial. Using a post-hoc power analysis, the study sample size is adequately powered to detect a difference of 0.3 between the two groups in incidence of poor psychological outcomes at 90% power and an alpha value of 0.05; an effect size that is considered small using Cohen’s standardized effect size criteria [59] Secondly, the study used objective and valid instruments to assess the different outcomes. Thirdly, this is one of few studies to assess the effect of different glycaemic treatment targets on mental health outcomes in women with GDM. Additionally, the results of this study are generalisable for use in healthcare settings managing women with GDM in New Zealand, as the study recruited hospitals nationwide. However, the study is specific to the New Zealand population of women with GDM and healthcare context, and whilst likely generalisable to women with GDM in similar healthcare settings may not be to those in low- and middle-income countries.

The main limitation of our study is that women who did not participate in our study (did not consent to completion of questionnaires on mental health) may differ from those who did with regards to the outcome (e.g., may have been suffering from poor mental health) which may result in a selection bias. Secondly, we used self-reported measures to assess mental health outcomes in our study which are not considered as diagnostic gold standards. The EPDS cut-off used in our study has, however, been reported as similar in accuracy to clinical interviews which are considered as the gold standard for diagnosis of depression during pregnancy and in the postpartum period [60]. Although the SF-36 questionnaire has been validated for use in the New Zealand population [61], the EPDS and short-item STAI have not been validated for use across all ethnicities in the New Zealand population to determine the optimal cut-off points. Additionally, the tight glycemic targets used in this study [(fasting ≤ 5.0 mmol/L (≤ 90 mg/dl), 1-h postprandial ≤ 7.4 mmol/L (≤ 133 mg/dl); 2-h postprandial ≤ 6.7 mmol/L (≤ 121 mg/dl)] differ slightly from tight targets recommended in other settings, like the United States [(fasting < 95 mg/dL (< 5.3 mmol/L); 1-h postprandial < 140 mg/dL (< 7.8 mmol/L); 2-h postprandial < 120 mg/dL (< 6.7 mmol/L)] [39].

## Conclusion

In summary, we found no difference in maternal mental health outcomes, namely anxiety, depression, and health-related quality of life, measured at 36 weeks’ gestation and 6 months after birth among women with GDM treated with tighter recommended glycaemic treatment targets compared to the previously used less tight glycemic targets in New Zealand. These findings suggest adoption of tighter glycaemic treatment targets in GDM care does not appear to benefit nor harm maternal mental well-being assessed at 36 weeks and 6 months after the birth.

## Data Availability

The data and materials used in this study are available from the corresponding author upon request. Data and associated documentation are available to users under the data sharing arrangements provided by the Maternal and Perinatal Research Hub, based at the Liggins Institute, University of Auckland. Proposals should be directed to researchhub@auckland.ac.nz.

## Abbreviations

aMD: Adjusted Mean Difference
aRR: Adjusted Relative Risk
BMI: Body Mass Index
CI: Confidence Interval
EPDS: Edinburgh Postnatal Depression Scale
GDM: Gestational Diabetes Mellitus
HRQoL: Health-Related Quality of Life
MCS: Mental Component Summary
OGTT: Oral Glucose Tolerance Test
PCS: Physical Component Summary
SAS: Statistical Analysis System
SF-36: 36-Item Short-Form General Health Survey
STAI: Spielberger State-Trait Anxiety Inventory
